# Stress‐Induced Melting Controlled Failure Mechanisms of Methane Hydrate

**DOI:** 10.1002/advs.202518367

**Published:** 2025-10-31

**Authors:** Yanlong Li, Yuan Zhou, Zhengcai Zhang, Minhui Qi, Yajuan Zhang, Yujing Jiang, Yunkai Ji, Qingguo Meng, Yongfu Sun, Nengyou Wu

**Affiliations:** ^1^ Laoshan Laboratory Qingdao 266237 China; ^2^ Key Laboratory of Gas Hydrate Ministry of Natural Resources Qingdao Institute of Marine Geology Qingdao 266237 China; ^3^ Graduate School of Engineering Nagasaki University Nagasaki 852–8521 Japan; ^4^ China National Deep Sea Center Qingdao 266237 China

**Keywords:** failure mechanisms, gas hydrate, nanoscale, pressure melting

## Abstract

Methane hydrate, a kind of nonstoichiometric crystalline, attracted worldwide attentions as a promising substitute energy. Its Dissociation is thought to be dominated by thermodynamic conditions, yet its intrinsic deformation behavior remains elusive, notably at the nanoscale. Here, substantial regional melting is found during nanoindentation on methane hydrate, supported by both molecular‐scale dynamic simulations and mesoscale low‐field nuclear magnetic resonance signals, revealing previously unknown failure mechanisms of methane hydrate. Stress‐induced regional melting is also identified to form a short, dumb, and multivariant “least resistance path” for the nanoindentation tip to penetrate into methane hydrate, distinct from hexagonal ice. Results confine the elastic modulus of methane hydrate to 9.8 ± 0.6 GPa, similar in orders of magnitude to hexagonal ice (12.8 ± 1.0 GPa) under 173.15 K. However, its hardness is 261.4 ± 24 MPa, nearly half that of hexagonal ice (526.6 ± 62 MPa). The discovery challenges the prevailing wisdom that elastic modulus reliably predicts hydrogen‐bonding phase‐reversible crystals, and asserts that hexagonal ice is a poor proxy for methane hydrate, at least from the mechanical perspective. The findings also enlighten new clues to model the stability of ice‐contained methane hydrate settings in high‐latitude hydrate provinces, along with palaeo‐methane‐release capacity during glaciation‐to‐interglaciation transitions.

## Graphical abstract

We study methane hydrate via nanoindentation and nuclear magnetic resonance (NMR), finding its regional melting and mechanical properties, challenging existing views on hydrogen‐bonded crystals.

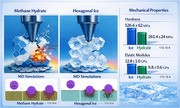

## 1 Introduction

Methane hydrate is a crystal formed via the combination of hydrogen‐bonding (to form caged structures from water molecules) and van der Waals forces (to enwrap methane molecules into the caged structures).^[^ ^1^ ^]^ The nucleation and formation mechanisms of methane hydrate have been extensively explored in previous researche.^[^ ^2^, ^3^ ^]^ Natural‐occurrence and ubiquitous‐distribution nature of methane hydrate on our planet (mostly in marine sediment and permafrost) and beyond enlightened studies on its significance for low‐carbon substitute energy,^[^ ^4^ ^]^ submarine geohazards,^[^ ^5^ ^]^ climate change,^[^ ^6^, ^7^ ^]^ deepsea biological chains,^[^ ^8^ ^]^ as well as the planet's habitability.^[^ ^9^ ^]^ Mechanical destabilization of methane hydrate is a crucial objective, as it would address extraordinary issues related to not only initiation of submarine geohazards (e.g., landslide, mud volcano)^[^ ^10^, ^11^ ^]^ and reservoir stability during artificial gas extraction,^[^ ^12^ ^]^ but also buffering from global climate change by valving methane emissions from deep‐sea geological settings.^[^ ^13^, ^14^ ^]^ When geological structures or external environmental conditions change, leading to an increase in loading on gas hydrates, it may trigger pressurized melting. This process of pressurized melting can result in mechanical weakening. Mechanical weakening propagates through hydrate‐bearing sediments, nucleating failure planes that may evolve into large‐scale landslides. Numerous efforts have been devoted to unveiling the mechanical properties of methane hydrate via either numerical simulations^[^ ^15^ ^]^ or experimental tests,^[^ ^16^ ^]^ whereas direct measurement of the deformation properties of methane hydrate from the nanoscale remains challenging,^[^ ^15^ ^]^ if not impossible. The practical difficulties in direct measurements for methane hydrate (partly resulted from the difficulties of remaining methane hydrate stable under an ambient‐pressure environment), combined with its ice‐analogous crystallographic properties, provoked the prevailing thought of taking ice as a substitute when exploring the physical properties of methane hydrate.^[^ ^17^ ^]^ For example, results from classic molecular dynamic simulations reveal that both methane hydrate and hexagonal ice exhibit elastic behavior and followed by brittle failure under uniaxial loading.^[^ ^15^, ^18^ ^]^ Furthermore, generation of some special ice structures (e.g., ice XVI, ice XVII, and cubic ice *I*_c_) by degassing gas molecules from hydrate^[^ ^19^, ^20^, ^21^ ^]^ seems to strengthen the similarities between ice and hydrate. However, there is no conclusive and convincing consensus on whether methane hydrate is replaceable by ice, since differences between ice and methane hydrate were also highlighted in many researches (e.g., Ref. [16, 22]).

**Figure 1:**
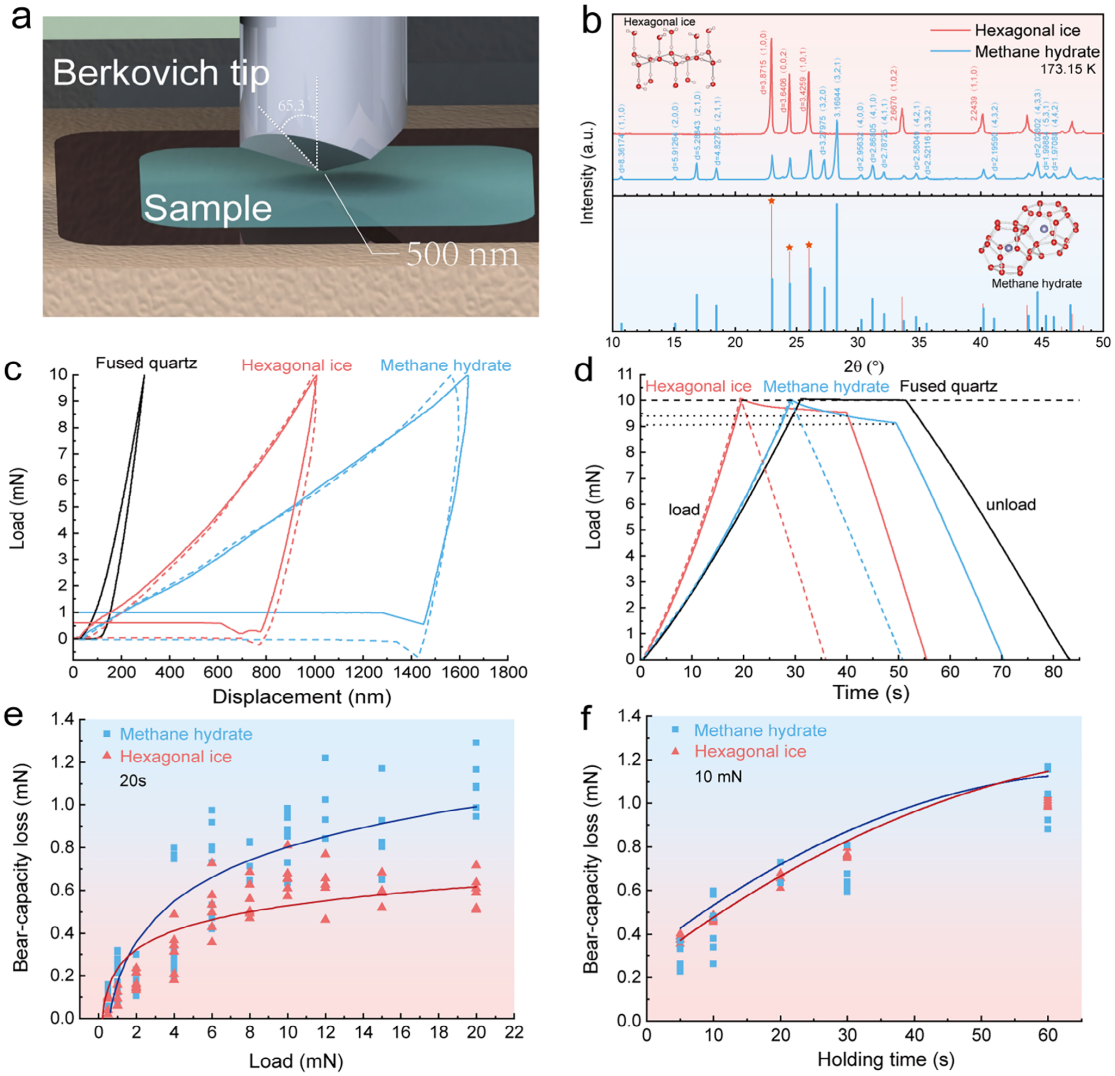
Nanoindentation device and typical indentation results. a) Schematic view of the Berkovich tip for nanoindentation. Diameter of Berkovich tip is 500 nm, and angle between the central axis and prism surface is 65.3°. b) Powder X‐ray diffraction results for the experimental samples of ice (red line) and hydrate (blue line). The methane hydrate samples belonged to the cubic crystal system, the space group was *Pm3n*, and the lattice constant *a* was ≈11.825 × 10^−10^ m, all of which were typical type I hydrates. In the process of PXRD tests, water vapor may condense and mix into the sample, so in addition to methane hydrate, the diffraction spectrum of the methane hydrate sample also contains hexagonal ice diffraction peaks. c) Typical displacement‐load curves for fused quartz (293.15 K), hexagonal ice (273.15 K), and methane hydrate (273.15 K). The solid lines were obtained following the international standard ISO 14577‐1. A 20 s holding stage is applied before withdrawl of the Berkovich tip. The dashed lines were obtained under the condition of immediate pave‐out of the Berkovich tip once it reached the maximum penetration depth. d) The indentation time‐load curves corresponding to the displacement‐load curves are shown in (c). Substantial force losses were observed during the holding stage for hexagonal ice and methane hydrate. e,f) the influence of indentation load and holding time on the force losses during holding at peak load. The fitting method was using a least squares regression based on the mean values from the experimental samples. The force loss values for methane hydrate (illustrated with a blue square) exhibit a more scattered tendency than those of hexagonal ice (illustrated with a red triangle). Compared to the fully occupied methane hydrate, the XRD results exhibit distinctive characteristic peaks indicative of a deviation from the fully filled state. The emergence of these peaks may potentially account for the dispersion observed in the data obtained from nanoindentation tests.

The long‐standing debates on whether methane hydrate can be substituted by ice encouraged us to develop an ultra‐low temperature nanoindentation device to unveil the intrinsic mechanical mechanisms of methane hydrate, as well as ice. The device is able to cool the sample to 113 K. This is the first attempt to directly measure the deformation behavior of methane hydrate via nanoindentation, to the best of our knowledge. A standardized Berkovich tip (**Figure** **1**a) is equipped in the device with a controllable strain accuracy of 1.0 nm and a maximum loading capacity of 200 mN. Methane hydrate was synthesized in a stainless high‐pressure vessel with methane (purity of 99.99%) and deionized water under the temperature of 274.65 K, whereas the ice was formed at ambient pressure under 253.15 K (see Experimental Section). The sample was then transported onto object panels of the nanoindentation device for further tests. Both ice and methane hydrate samples were structurally characterized via powder X‐ray diffraction (XRD) before indentation, which confirms the structure‐I‐type hydrate, and hexagonal ice *I*_h_ (Experimental Section and Figure 1b). A minor ice signal is detected in the methane hydrate sample, which is ascribed to the unavoidable sample pollution during sample transfer for XRD tests. The reliability and repeatability of the nanoindentation device are verified via repeated tests for standardized fused quartz, hexagonal ice, and methane hydrate (see Figure S1, Supporting Information for details). It is noting that fused quartz was accepted as a standard reference material to verify the reliability of nanoindentation.

## 2 Results

### 2.1 Load‐Holding Leads to Bearing‐Capacity Loss for Methane Hydrate

Figure 1c compares the Berkovich indentation curves for standardized fused quartz, hexagonal ice, and methane hydrate under the same load, in which the solid lines were obtained by following the international standard ISO 14577‐1. The indentation mode for fused quartz complies well with the available data,^[^ ^23^ ^]^ strengthening the reliability of the device. The maximum penetration depth for methane hydrate approaches 1634 nm under the load of 10 mN, whereas the maximum penetration depth for hexagonal ice is ≈1010 nm. It is apparent that methane hydrate is much softer, manifested by deeper indentation depth under the same loading, than hexagonal ice.

A particularly surprising finding is that the apparent load cannot return to the zero point during unloading for either hexagonal ice or methane hydrate. The fact that the result for fused quartz goes as expected enlightens us that the deviation might be an intrinsic mechanical response of the hexagonal ice and methane hydrate. The insight provoked puzzling inconsistencies between the results for fused quartz and phase‐reversible crystalline (i.e., hexagonal ice and methane hydrate). To understand the origin of such discrepancies, we checked the time‐load curves during indentation (Figure 1d), observing unforeseen substantial load decrease (i.e., force loss) during the 20 s holding at peak load for methane hydrate and hexagonal ice, in striking contrast with fused quartz. This implies deterministic bearing‐capacity loss under loading for methane hydrate and hexagonal ice. We further correlate the force loss with the loading value and holding duration (Figure 1e,f), revealing that the bearing‐capacity loss for both hexagonal ice and methane hydrate increases logarithmically with the increase in either loading or holding duration, while the data for methane hydrate exhibit a more scattered tendency than hexagonal ice. The higher penetration depth under the same loading (Figure 1c), combined with the more scattered force loss connected to load‐holding (Figure 1e,f), leads us to conclude that while both materials exhibit stress‐induced melting, methane hydrate's deformation is dominated by cage‐structure collapse at the molecular scale, in contrast to hexagonal ice's dislocation‐mediated plasticity.

### 2.2 Stress‐Induced Regional Melting Dominates the Failure of Methane Hydrate

**Figure 2:**
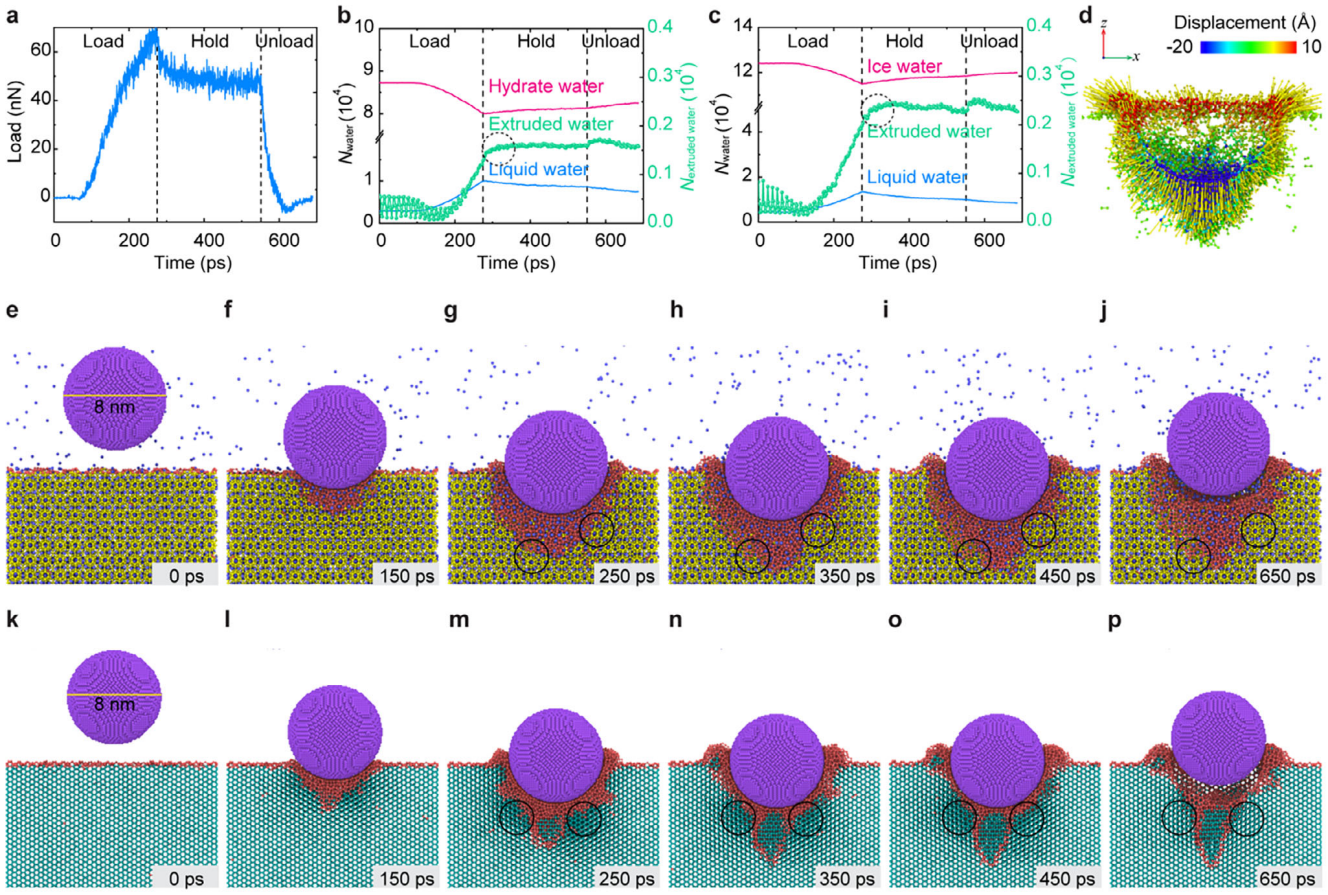
Indentation mechanisms analyzed by molecular dynamics simulations. a) Typical indentation time‐load curves for monocrystalline methane hydrate from molecular dynamic simulation. The time‐load curve is consistent well with experimental results, replicating the substantial force loss during holding at peak load. b) The evolutionary of the number of hydrated water molecules (pink line), melted water molecules (blue line), and extruded water molecules (green line) during the indenting of methane hydrate. The extruded water molecules were defined to be the liquid water molecules that dissipate above the original hydrate surface. c) The evolutionary of the number of iced water molecules (pink line), melted water molecules (blue line), and extruded water molecules (green line) during indenting hexagonal ice. The extruded water molecules were defined to be the liquid water molecules that dissipate above the original ice surface. d) Displacement field of water molecules surrounding the indentation crater during holding. e–j) Snapshots taken from various stages of indentation for methane hydrate, where (e) represents the moment when the tip approaching to the surface of the sample. f–h) are the indenting process. The position of the tip remains the same in h,i), representing the load holding at the peak load. (j) the unloading process. k–p) Snapshots taken from various stages of indentation for monocrystalline hexagonal ice. The purple ball in (e–p) represents the tip of the Berkovich tip. Dark‐red areas surrounding the indentation crater represent the melted methane hydrate and/or melted hexagonal ice under the effect of indentation. Discrete blue dots above the solid surface in (e–j) are the methane molecules that escaped from the melted methane hydrate.

Molecular dynamic simulation (see Experimental Section, **Figure** **2** and Movies S1–S4, Supporting Information) aids the understanding of the indentation mechanisms. The simulation results replicate the substantial force loss during holding at peak load (Figure 2a), complying well with the results from indentation tests (Figure 1d). Interestingly, we observe notable regional melting, inferred from time‐dependent increase of the liquid water molecules (or decrease of the hydrate water molecules) of the systems (Figure 2b,c), once the indenter contacts and penetrates into the methane hydrate and/or hexagonal ice samples. Snapshots taken from various stages of the simulation display the propagation of the melting area during penetration. The propagation of the melting area beneath the indenter preceded with the insertion of the tip, forming a “least resistance path” (Figure 2f–h and l–n) for the penetration of the indenter. This is quite astonishing since such a melt‐induced “least resistance path” is in clear contrast with the conventional crystal deformation, in which the indenter propagates following the shear band at the crystal boundary interfaces.

Comparative analyses indicate that the shapes of regional melting are different between hexagonal ice and methane hydrate, which signifies diverse failure mechanisms. In methane hydrate, a short, dumb, and multivariant regional melting area is observed surrounding the indenter as the indenter paves into the samples. However, the “least resistance path” in hexagonal ice exhibits an elongated, streamlined, and single‐variant shear fault at the tip‐direction, which could be interpreted as stress‐induced quasi‐plasticity or amorphization^[^ ^24^ ^]^ of the crystalline structures. This implies that the indentation failure of methane hydrate is dominated by regional melting, whereas microcracking appears to overwhelm the influence of regional melting in hexagonal ice. As a result, the findings somehow prove that hexagonal ice is more prone to brittle failure, whereas methane hydrate is prone to more pronounced viscoelastic behaviors.

Furthermore, the differences in deformation mechanisms, which can somehow be attributed to host–guest molecule interactions.^[^ ^25^ ^]^ Raman spectroscopy measurements indicate that the cage occupancy of laboratory‐synthesized methane hydrate ranges between 89.8% and 95.8%, independent of experimental conditions.^[^ ^26^ ^]^ The presence of methane molecules in the cage structure is uncertain, which provides an acceptable explanation for the experimental results that bearing‐capacity loss (Figure 1e,f) for methane hydrate is much more scattered than hexagonal ice during holding. It is noted that the aforementioned simulations were conducted on monocrystalline methane hydrate and hexagonal ice samples. However, similar indentation‐induced failure processes are also observed for polycrystalline methane hydrate and hexagonal ice (see Figure S3 and Movies S3 and S4, Supporting Information), confirming the reliability of the results.

### 2.3 Dissipation of Melted Water Molecules Results in Internal Structural Relaxation

Theoretically, bearing‐capacity loss during load‐holding can be traced into the time‐dependent internal relaxations of materials, and is always interpreted as creep.^[^ ^27^ ^]^ The aforementioned regional melting is a possible explanation for internal relaxations of methane hydrate and hexagonal ice. However, the number of hydrate water molecules increases during holding (see magenta‐colored line in Figure 2b), indicating the termination of stress‐induced regional melting and initiation of recrystallization once the penetration of the indenter is suspended. Recrystallization would somehow enhance the surface of the system and prevent further internal relaxation, and hence, regional melting is unlikely the inducement for bearing‐capacity loss during holding.

We further analyzed the flow field of the melted water within the indentation crater (Figure 2d), observing that liquid water molecules at the contacting faces and edges fluctuate and escape upward from the indentation crater, forming a ring‐like liquid cluster surrounding the indentation crater. The anisotropic dissipation tendency of liquid water molecules is a combined result of the steric constraints in the lateral direction and the thermodynamic preferences. The dissipating water molecules recrystallize partially and entail realignment of the system structure. Such a water‐dissipation‐induced force loss can also be characterized quantitively by the number of water molecules extruded out from the indentation crater (see green solid lines in Figure 2b,c). Therefore, we assert that water dissipation and structural realignment occur simultaneously during holding, resulting in internal relaxation and expressed as bearing‐capacity loss.

The internal relaxation might result in a nonideal, defect indentation surface and lead to fading of the intrinsic mechanical parameters of methane hydrate. Therefore, our results challenge the international standard ISO 14577‐1, namely that the load‐holding should be removed from the test schedules to obtain intrinsic hardness and elastic modulus for phase‐reversible crystalline such as methane hydrate and hexagonal ice (see dotted lines in Figure 1d).

### 2.4 Recrystallization Induces a Mechanical “Memory Effect”

Another intriguing phenomenon is the unforeseen recrystallization of methane hydrate after the withdrawal of the indenter (i.e., during unloading, circled in Figure 2i,j). Such a notable recrystallization might be accompanied by restorable deformation and reversible mechanical property changes. Here, we interpret the recrystallization as a mechanical “memory effect” for methane hydrate, in comparison with the memory effect found during hydrate nucleation processes.^[^ ^28^ ^]^ This effect fundamentally differs from classical elastic recovery, as evidenced by the time‐dependent depth recovery and reduced residual depth upon reloading, indicating structural reorganization through local cage collapse and recrystallization. The result somehow proves that methane hydrate can be categorized into shape memory crystals with restorable deformations under stress. Similar recrystallization was also observed in hexagonal ice (Figure 2o,p), albeit the recrystallization position differs from that of the methane hydrate.

**Figure 3:**
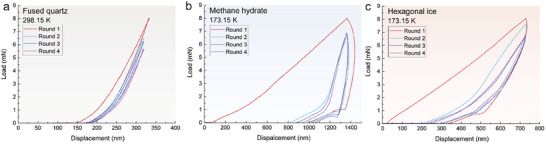
Repeated indentation curves for fused quartz a), methane hydrate b), and hexagonal ice c). The first round of indentation was done under the load of 8.0 mN, identical to the dashed lines shown in Figure 1c,d. Once the indenter is reverted back to the zero point, another immediate penetration into the same indentation crater is initiated under the displacement‐controlling mode. Repeat the penetration action for three rounds for each sample. That is, during the subsequent indentation tests, the maximum penetration depth and penetration rate were set to be equivalent to the first‐round test, while measuring and recording the load during penetrating and retrieving the indenter. (a) the penetrating curves overlaps the retrieving curves for fused quartz, indicating there is no plastic deformation during the subsequent indentation test. (b) obvious lagging of the retrieving curves can be observed, compared to the penetrating curves during the 2nd–4th rounds of penetration tests. This proves that the existence of plastic deformation, and hence, proves the existence of mechanical memory effect. The area under the penetrating‐retrieving curves shrinks with the increase in test rounds, indicating the fading tendency of mechanical memory effect. (c) similar lagging tendencies are observed during the 2nd–4th rounds of penetration tests for hexagonal ice.

Further indentation tests were conducted to confirm the existence of the mechanical memory effect for methane hydrate (see **Figure** **3**). The indenter was penetrated into the same depth at the same point for several rounds successively, with the same penetration rate. Theoretically, the deformation of the sample consists of plastic deformation and elastic deformation. The plastic deformation would vanish during the subsequent testing rounds, supposing there is no mechanical memory effect. As a result, the unloading curves shall overlap the loading curves during the subsequent testing rounds, as was shown for the fused quartz (Figure 3a). Here we find substantial plastic deformation during the subsequent testing rounds for both methane hydrate and hexagonal ice, labeled by the area covered by the loading‐unloading envelopes (Figure 3b,c). The finding confirms the mechanical memory effect for both methane hydrate and hexagonal ice, consistent with the prediction from molecular dynamic simulations, albeit with substantial load loss on the displacement‐load curves among different testing rounds. Besides, it is noted that the area covered by the loading–unloading envelopes for both methane hydrate and hexagonal ice is shrinking with the increase of testing rounds, indicating the fading of the mechanical memory effect.

### 2.5 Methane Hydrate is Much Softer Than Hexagonal Ice at 173.15 K

**Figure 4:**
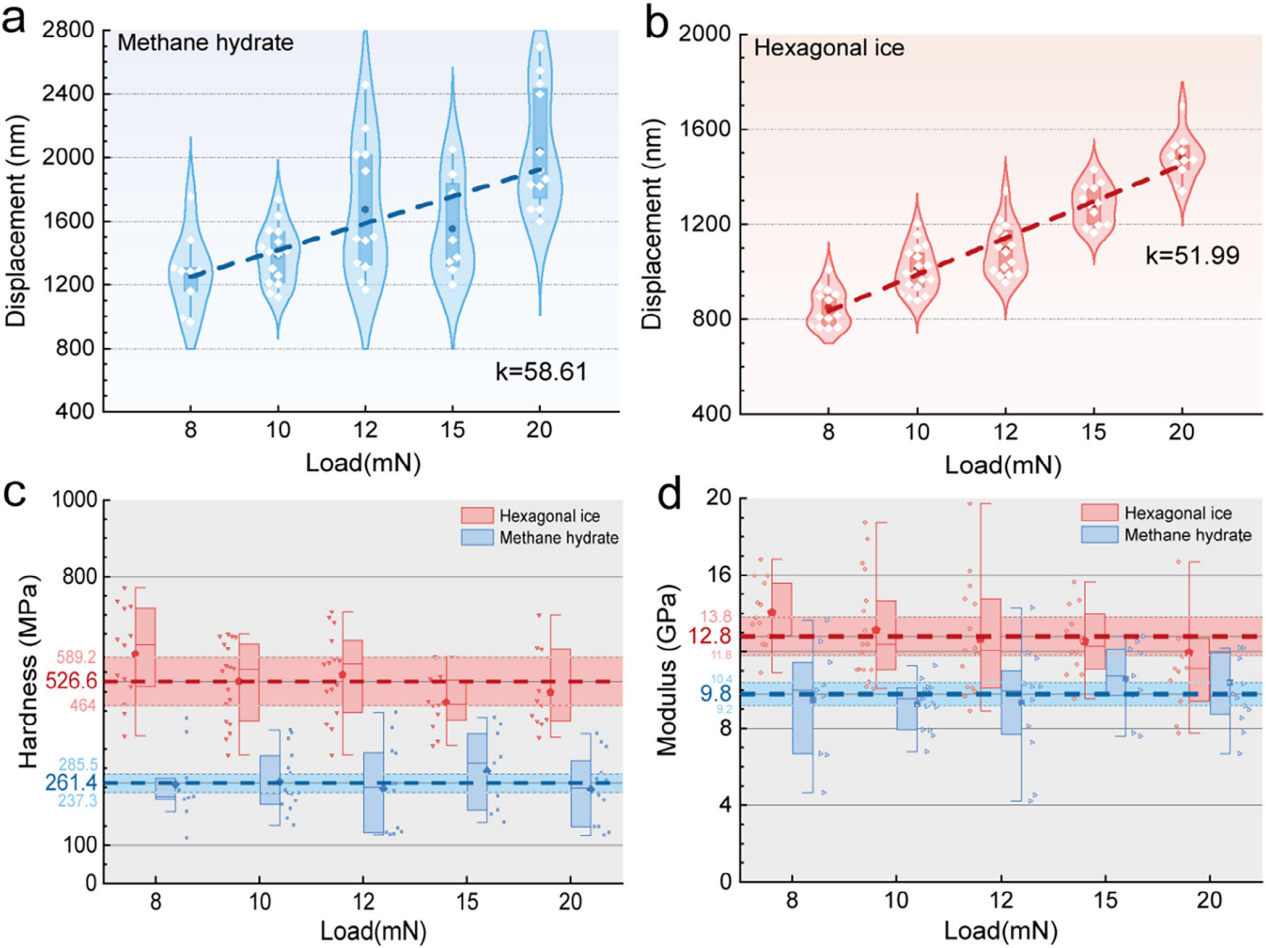
Intrinsic mechanical properties of methane hydrate and hexagonal ice. a) Relationship of the maximum indentation depth versus load for methane hydrate. The maximum indentation depth increases linearly from 1279 to 2041 nm, with an increasing rate of *k*_MH_ = 58.61 nm mN^−1^, when the load increases from 8 to 20 mN. b) Relationship of the maximum indentation depth versus load for hexagonal ice. The maximum indentation depth increases linearly from 850 to 1482 nm, with an increasing rate of *k*_HI_ = 51.99 nm mN^−1^, when the load increases from 8 to 20 mN. c) Comparison of the hardness for methane hydrate and hexagonal ice, indicating that the hardness of hexagonal ice is almost two times of that for methane hydrate. d) Comparison of the elastic modulus for methane hydrate and hexagonal ice, displaying they lie in the same orders of magnitude.

Hardness is one of the most important intrinsic mechanical properties, however, is less straightforward to identify unless the texture of the sample is homogeneous. **Figure** **4**a,b exhibit the maximum penetration depth of the Brkovich tip under different loading for hexagonal ice and methane hydrate, respectively. The dispersion of indentation displacement data for methane hydrates, as observed in the relationship between indentation displacement and load (Figure 4a,b), contrasts with the more concentrated data for hexagonal ice. This discrepancy is attributed to the occupancy rate of cages in methane hydrates, where not all cages are filled with methane gas, leading to heterogeneity in the hydrate. Conversely, the crystal structure of hexagonal ice is more stable. The maximum penetration depth for methane hydrate increases linearly from 1279 to 2041 nm, when the load increases from 8 to 20 mN. The maximum penetration depth is well below 10% of the thickness of the samples (i.e., 0.3 cm), while thicker over ten times than the available maximum thickness of the quasi–liquid layer (i.e., less than 100 nm^[^ ^29^ ^]^) on the hydrate surface. In comparison, the maximum penetration depth of hexagonal ice increases linearly from 850 to 1482 nm when the load increases linearly from 8 to 20 mN, although we also observe limited fluctuations of the maximum penetration depth under the same loading. The linear increasing trend of the maximum penetration depth is a directive indicator that the texture of the samples is homogeneous, whereas the limited fluctuations under the same loading can be ascribed to the randomness in sample preparation and unavoidable test errors. Hence, we could derive intrinsic mechanical properties for both hexagonal ice and methane hydrate via the indentation tests, such as hardness.

For comparison, we render the hardness of methane hydrate and hexagonal ice as functions of load. Our experiment defined the hardness of methane hydrate to 261.4 ± 24 MPa (Figure 4c), which is only half of that for hexagonal ice (526.6 ± 62 MPa). Although the mechanical properties of methane hydrate have been repeatedly‐stated in the previous studies via various approaches, our results appear to be the first direct measurement of intrinsic mechanical parameters for methane hydrate at the nanoscale, especially the hardness. The results prove that the hexagonal ice is much harder than methane hydrate, although the current indentation test is unable to consider the dependence of temperature. The result challenges the prevailing choice of taking ice as a substitute when examining the physical properties of either massive methane hydrate or hydrate‐bearing sediment. The mechanical contrast between hydrate and ice may intensify at warmer conditions, where thermal effect promotes the hydrogen‐bonding dynamics and lattice stability. Systematic temperature‐dependent studies are warranted in the future to quantify this effect.

### 2.6 Elastic Modulus for Methane Hydrate

The indentation‐derived elastic modulus for methane hydrate is 9.8 ±0.6 GPa (Figure 4d) at 173.15 K, in excellent agreement with the previous results from classic molecular dynamic simulation (7.68–9.71 GPa)^[^ ^15^ ^]^ and first‐principles calculation (11.07 GPa).^[^ ^30^ ^]^ It is imperative to emphasize that the previous contactless experiment (e.g., high‐pressure Brillouin spectroscopy) has defined the elastic modulus of methane hydrate into the range of 3.5–17 GPa.^[^ ^31^ ^]^ In relation to the contactless experiments, our results reduce the data divergence significantly and narrow the gap between experimental and theoretical predictions. It is noted that the previous centimeter‐scale uniaxial compression for massive methane hydrate (identified as polycrystalline hydrate) obtained from an authentic deep seabed (i.e., naturally formed) attributed the elastic modulus of methane hydrate to 300 MPa,^[^ ^32^ ^]^ ≈1/33 weaker than the indentation‐derived values. The inverse Hall–Petch effects provide a possible explanation for the discrepancies,^[^ ^15^ ^]^ but a more feasible possibility is that the naturally formed methane hydrate contains other matters (e.g., mud, carbonate) and/or small cracks, albeit not visible under microfocus X‐ray Computed Tomography images. Therefore, the realization of direct, contact measurement of intrinsic mechanical properties for methane hydrate at nanoscale provides an opportunity of bonding physical experiments and molecule‐scale simulation results for ideal, non‐defective methane hydrate, and might be extended to other metastable phase‐reversible crystals.

Besides, the elastic modulus of the hexagonal ice (i.e., 12.8 ± 1.0 GPa, Figure 4d) is higher than methane hydrate slightly at 173.15 K, yet lies in the same orders of magnitude as methane hydrate. Notably, when compared at equivalent supercooling (20 K), methane hydrate (9.8 ± 0.6 GPa at 173.15 K) exhibits nearly an order‐of‐magnitude higher modulus than hexagonal ice (1.21 ± 0.26 GPa at 253.15 K).^[^ ^33^ ^]^ demonstrating that the mechanical differences stem primarily from their distinct lattice architectures rather than thermal history. The observed mechanical difference likely stems from methane occupation in clathrate cages, which stabilizes the hydrogen‐bond network against deformation compared to pure ice's hexagonal symmetry.^[^ ^34^ ^]^

## 3 Discussion

### 3.1 Mechanical Contrasts Between Methane Hydrate and Hexagonal Ice

The realization of direct, contact measurement of intrinsic mechanical properties via nanoindentation provides an opportunity for bonding physical experiments and molecular‐scale simulation results for methane hydrate, as well as for other metastable crystals. The immediate implications from these results are that both the failure mechanisms and hardness of methane hydrate and hexagonal ice are completely different, albeit they share similar phase‐reversible characteristics during indenting. The distinct mechanical responses may originate from fundamental structural differences between hexagonal ice and methane hydrate. The structural differences between methane hydrate and hexagonal ice mainly result from the presence of methane molecules. The clathrate structure and its occupancy rate might be the reason for the different mechanical properties of methane hydrate and hexagonal ice. Differences of the failure mechanisms between hexagonal ice and methane hydrate are much more significant than presently supposed, strengthening that hexagonal ice is a poor proxy for methane hydrate, at least from a mechanical perspective. The substantial differences in their deformation mechanisms arose a very interesting possibility to tune the deformation mechanisms of ice via hydrate doping. In authentic geological settings (e.g., in the glacially‐influenced continental shelves), ice‐contained methane hydrate settings are widely distributed^[^ ^35^, ^36^ ^]^ in the high‐latitude hydrate provinces (e.g., Antarctic continental margin,^[^ ^35^ ^]^ offshore N Svalbard of the Arctic Ocean^[^ ^10^, ^37^ ^]^). We anticipate that the mechanical instabilities of the ice‐contained methane hydrate systems result from the competitive effect between hydrate and ice under these circumstances. Furthermore, the subglacial formation of methane hydrates can hinder “pore‐water piracy,” enhance basal traction, and act as sticky spots that regulate ice stream flow,^[^ ^38^ ^]^ the finding provides vital insights to qualitatively model the past and contemporary ice‐stream regulating the capacity of subglacial methane hydrate, and their influencing mechanisms on the potentially widespread subglacial landslides. So far, the ice‐to‐hydrate interfacial failure, along with their controlling mechanisms in the stability of either ice sheet or subglacial sediment, has not been recognized. Considering the approximately same level of elastic modulus but substantially different hardness and failure contributors for hexagonal ice and methane hydrate, the stability dynamics of hydrate‐contained ice streams and hydrate‐contained permafrost are more complex than previously thought.

### 3.2 Nuclear magnetic resonance (NMR) Evidence for Stress‐Induced Hydrate Melting

**Figure 5:**
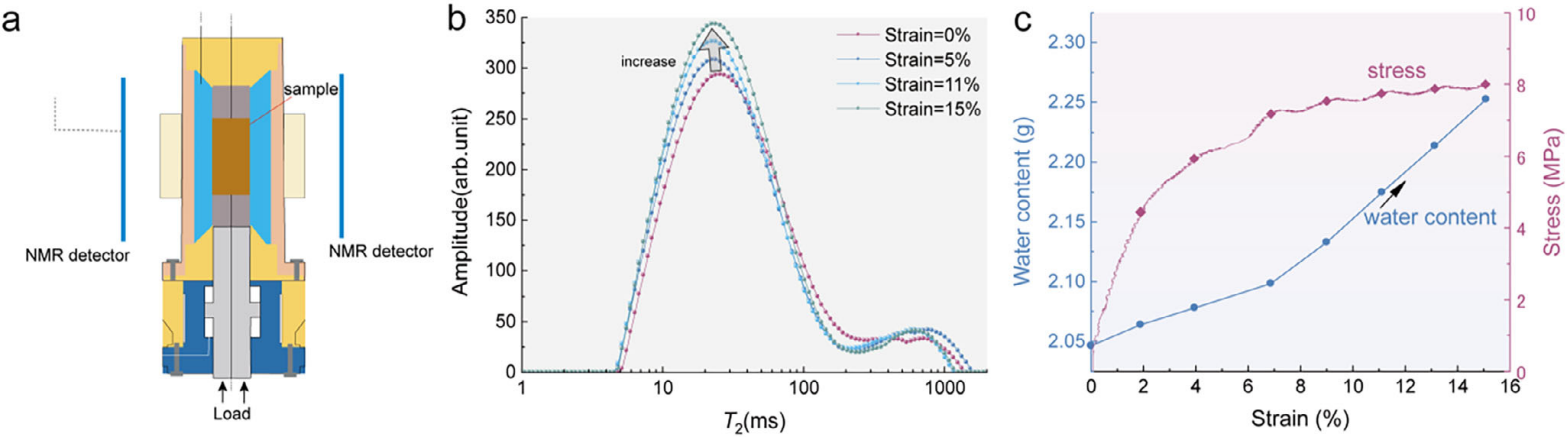
Joint test of triaxial shear and low‐field nuclear magnetic resonance (NMR). a) Schematic diagram illustrating the theory of joint test of triaxial shear and low‐field NMR for methane hydrate‐bearing sediment. The sample is 50 mm in length and 25 mm in diameter. The pore pressure and confining pressure is isolated by a perfluoro membrane, and the high‐pressure vessel is made from PEEK. Methane hydrate is synthesized via deionized water and methane gas in sandy sediment under a pore pressure of 7.0 MPa and a system temperature of 1.0 °C. The vessel is equipped on the panel of the low‐field NMR. *T*_2_ distribution is detected during axial loading. b)) Representative *T*_2_ distributions during triaxial shearing at a constant shearing rate of 0.2 mm min^−1^ and a constant effective confining pressure of 1.0 MPa. Downward shifting of the *T*_2_ curves with the increasing shear strain is direct evidence of hydrate melting. c) Comparison between the stress and overall water content within the sample under different strains. Water content is interpreted from the *T*_2_ distribution curves in b.

Furthermore, our results underscore melting‐controlled failure mechanisms of methane hydrate under circumstances of stress‐state alteration. The findings enlighten us with new clues for the stability prediction of methane hydrate under the circumstances of geological activities. However, some may argue how does the nanoscale findings affect meso‐to‐macro scale geobehaviors in realistic settings, in which methane hydrate is confined in sediment pores and thus forming hydrate‐bearing sediment. Here, we performed a combing test of triaxial shearing and low‐field NMR for methane hydrate‐bearing sandy sediment in mesoscale (see Experimental Section, sample size Φ25mm × 50 mm). A continuously increasing axial load is applied onto the sample with a constant shearing rate of 0.2 mm min^−1^, whereas the transverse relaxation time (*T*_2_) distribution is recorded simultaneously to explain overall water content evolution within the sample (**Figure** **5**a). A substantial increase in the peak amplitude of the *T*_2_ distribution (Figure 5b) is direct evidence of the increase of water content, most likely originating from methane hydrate melting as the sample is isolated from the water supply during shearing. Hence, the *T*_2_ distribution can be quantitatively interpreted into water content (Figure 5c), and nonlinear hydrate melting is proven to occur with increasing strain, although the current data is limited. This is the first laboratory observation, to be best of our knowledge, of stress‐induced melting while shearing hydrate‐bearing sediment, indicating the previously undetected failure mechanisms of hydrate‐bearing realistic geological settings. The stress relaxation observed in microscopic nanoindentation and the increase in liquid water content detected by macro‐scale NMR collectively confirm the phenomenon of pressure‐induced melting in methane hydrates. Furthermore, We hypothesize that this phenomenon during shear deformation is not exclusive to methane hydrate but may also occur in other structure‐I clathrate hydrates, such as carbon dioxide (CO_2_) hydrate. From a molecular perspective, CO_2_ hydrate shares the same sI cage structure with methane hydrate, consisting of hydrogen‐bonded water cages encaging small, nonpolar guest molecules. The stability of these hydrates is governed by the balance between van der Waals interactions of the guest molecules and the hydrogen‐bonding network of the host lattice. Under shear stress, local lattice distortions could similarly destabilize the cages, leading to dissociation.

### 3.3 Stress‐Induced Melting as an Underestimated Methane‐Release Triggering Mechanisms

The previous wisdom believed that bottom water temperature and sea level changes are the main contributors to methane hydrate stability.^[^ ^39^ ^]^ The rapid shift from a glacial period to an interglaciation increases the pore fluid pressure of the marine sediment, promoting conditions conducive to methane hydrate accumulation,^[^ ^6^, ^11^, ^40^ ^]^ thus the accumulation tendency and thickness of methane hydrate reservoir increase. Since the formation and thickening of the hydrate layer have been proven to filter and prevent the ascending rate of methane bubbles from the seafloor into the upper water column significantly,^[^ ^41^, ^42^ ^]^ possibly in the form of “crustal fingering,”^[^ ^43^ ^]^ the glaciation‐to‐interglaciation transition would theoretically mitigate the global atmospheric methane. However, our results from molecular‐scale simulation, nano‐scale indentation, and mesoscale shear test, imply that the overburden stress increase might result in considerable stress‐induced melting of methane hydrate, which provides a feasible explanation for the findings that rapid sedimentation triggered widespread methane hydrate dissociation during the end of the last glaciation offshore mid‐Norway.^[^ ^44^ ^]^ Under these circumstances, the occurrence of stress‐induced melting of methane hydrate would somehow regulate the methane emission processes. Consequently, the methane seepage prevention capacity of the hydrate layer might be weakened during glaciation‐interglaciation transitions, and hence, it is hypothesized that methane emission into the water column and atmosphere would be underestimated if the stress‐induced melting of methane hydrate was ignored.

## 4 Experimental Section

### Methane Hydrate Sample Preparation

The sample container was a top‐unenclosed cuboid‐hollow made from copper. The inner surface of the container was treated into hydrophobic and the outside surface was covered with a specially‐shaped acrylic. The inner size of the container was 7 × 7 mm in length and width, and 3 mm in depth. Five containers were placed into a high‐pressure vessel horizontally and evenly. Each of the containers was fully filled with deionized water and covered by a derased hydrophobic acrylic plate, prior to the sealing of the high‐pressure vessel. The process was followed by vacuum pumping of the high‐pressure vessel under ambient temperature. Afterward, the container‐vessel assembly was positioned into a low‐temperature water bath, cooled to 274.65 K, and pressurized with precooled pure methane (purity of 99.99%) to 5.0 MPa. The temperature‐pressure condition was remained for 72 h, and pure methane hydrate would be formed within the containers, and hence, ready for further transfer and tests. The derased hydrophobic acrylic plate ensured that the surface of the sample was in a relatively smooth state, which was profitable for the indentation test.

### Ice Sample Preparation

An ice sample was prepared within the same containers that were deployed during methane hydrate preparation. Ice sample preparation procedures also followed those for methane hydrate, except that ice was formed under ambient pressure under the temperature of 253.15 K.

### Powder X‐Ray Diffraction Characterization

Both the ice and methane hydrate were characterized using a Bruker D8 Advance Diffractometer. The X‐ray source of the diffractometer was a Cu anode with a test voltage of 40 kV and current of 40 mA, and the detector was a 1D LynxEye linear detector. All samples were tested between 2*θ* angles of 10° and 60°, with scan steps of 0.02° and data acquisition time of 1 s per step.

First, the samples were taken out from the containers and transferred into a grinding manger. It was noted that the grinding manger and grinding hammer, along with the test panel of the diffractometer, were precooled to 173.15 K prior to sample transfer. Then the samples were grinded in the nitrogen environment and transferred onto the test panel of the diffractometer for further characterization. During X‐Ray diffraction measurement, it was observed that the methane hydrate samples were polluted (inferred from the ice‐peaks in the methane hydrate samples, albeit tiny intensity) due to the unavoidable contact of the samples to atmosphere, although the entire sample transfer process took less than 1 min.

### Nanoindentation Measurement

The setup consists of a glovebox, a temperature control module, an indentation panel, a nanoindenter console, and a vibration isolator. The glovebox ensures a nitrogen environment at room‐temperature, whereas the temperature control module was able to control the temperature of the indentation panel down to 93.15 K, with a controlling accuracy of ± 2.5 K. The design avoids the impact of ultra‐deep temperature on wearing parts of the device, but enables us to cool both the test samples and indentation tip. The indentation tip was a standard Berkovich tip, with a diameter of 500 nm and central‐axis versus prism‐surface angle of 65.3°. The nanoindenter console motivates the Berkovich tip to collect the indentation data, with a controllable strain accuracy of 1 nm and a maximum loading capacity of 200 mN. The temperature control module, indentation panel, nanoindenter console were packaged onto the vibration isolator, and encircled by the glovebox.

For indentation measurement for methane hydrate, the glovebox was swept twice by nitrogen and then enter the circulation mode to keep the water content in the glove box below 0.1 ppm. Meanwhile, the indentation panel, along with the Berkovich tip, was cooled to 173.15 K. Synchronously, the high‐pressure vessel was placed into the liquid nitrogen environment and then vented. The sample container, along with sample, was transferred into an intermediate cup filled with liquid‐phase nitrogen, and moved into the glovebox. Subsequently, sample container was moved and positioned onto the indentation panel for further test. Therefore, the contact between the methane hydrate sample and atmospheric air was strictly excluded during the sample transfer and indentation test. At least 50‐rounds of indentation measurements were conducted on the same sample, and the distance between each two indenting points was set between 5 and 10 µm. The data acquisition frequency for penetration depth and real‐time load was 50 Hz in each indenting round. Furthermore, the hardness (*H*) was calculated using the Oliver–Pharr method (Oliver & Pharr, 1992): *H* = *P*_max_/A_c_, where Pmax is peak indentation load, A_c_ is real‐time projected contact area, continuously corrected for pile‐up/sink‐in effects via:

(1) $$A_{c}=24.5{\left(h_{\max}-\varepsilon \frac{P_{\max}}{S}\right)}^{2}$$

where *h*_max_ is the max depth of the indenter, *S* is the slope of the unloading curve.

The sample transfer procedures and indentation methods for ice were similar to that for methane hydrate, except that the operation for ice is conducted under atmospheric pressure, hence venting is not required.

### Molecular Models and Computational Configurations

The coarse‐grained potentials^[^ ^45^ ^]^ were employed to model water and methane molecules. The indenter was set to be a rigid body, while the interactions between nanolaminate with indenter were represented by the Lennard Jones potentials, $\phi_{LJ}\left(r_{ij}\right)=4\varepsilon \;\left[{\left(\frac{\sigma }{r_{ij}}\right)}^{12}\;-\;{\left(\frac{\sigma }{r_{ij}}\right)}^{12}\right]$ (*r* < *r*_c_), where ε_*methane* − *C*_ = 0.125 kcal mol^−1^, ε_*water* − *C*_ = 0.106 kcal mol^−1^, σ_*methane* − *C*_ = 0.3565 nm, and σ_*water* − *C*_ = 0.3283 nm. The distance cut‐off value is set as 0.7 nm.

The single‐crystal structures of hexagonal ice and methane hydrate were created using the GenIce code. Thereafter, a supercell of 14 × 14 × 10 unit cells of methane hydrate was generated for the monocrystalline configuration, while 12 × 12 × 7 unit cells for the ice configuration. The polycrystals were obtained using the ATOMSK software package, which employs a Voronoi construction based on the aforementioned single‐crystal unit cells as seeds. The resulting polycrystals have a volume of 16 × 16 × 16 nm^3^ and comprise 12 Voronoi grains with random geometry. These configurations were employed as nanolaminates.

### Molecular Simulations

The simulations were conducted using the large‐scale atomic/molecular massively parallel simulator (LAMMPS). The nanolaminates were initially minimized in energy via the conjugated gradient method and subsequently relaxed under the *NpT* ensemble (constant number of particles, pressure, and temperature) for 5 ns at 173 K and zero pressure, utilizing a Nosé–Hoover barostat and thermostat. The equations of motion were integrated with a time step of 5 fs. Afterward, a vacuum space with a width of 12 nm was incorporated into the nanolaminate along the *z*‐axis. The nanolaminates were then subjected to a further 5 ns of equilibrium under the *Np_x_p_y_T* ensemble. Subsequently, the nanolaminates were divided into three distinct layers along the *z*‐direction, designated as a fixed layer, a thermostat layer, and a Newtonian layer, respectively. The fixed layer and the thermostat have a width of 1 and 2 nm, respectively, while the remaining was the Newtonian layer. Then, a spherical diamond indenter with a radius of 4 nm was positioned on the nanolaminate with a 1.5 nm offset. Finally, nanoindentation simulations were conducted at 173 K by pressing the indenter into the substrate with a constant indenter velocity of 0.02 nm ps^−1^. During the nanoindentation process, the force and velocity of atoms in the fixed layer were set to zero, while the Langevin thermostat was applied to the thermostat layer. Once the indenter had reached a depth of 4 nm, the holding and unloading processes were initiated. The water molecules within the nanolaminate were classified using the CHILL+ code, which was integrated into the OVITO package.

### Triaxial Shear and Low‐Field NMR Test

A mini high‐pressure vessel was developed for the triaxial shearing test of hydrate‐bearing sediment (Patent No. CN111289553B), with a sample size of Φ25mm × 50 mm.^[^ ^46^ ^]^ The vessel, made from PEEK, can withstand a maximum inner pressure of 20.0 MPa under a triaxial stress state, and can be installed onto the test panel of a low‐field nuclear magnetic resonance analyzer. The vessel was filled with a mixture of deionized water and silicon sand (diameters range from 120 to 180 meshes), and pressurized to 6.0 MPa to form hydrate under the temperature of 2.0 °C and effective confining pressure of 1.5 MPa for over 72 h. Afterward, undrained triaxial shear was performed by applying axial load to the sample at a constant shearing rate of 0.2 mm min^−1^. The axial load, together with real‐time *T*_2_ distribution was recorded simultaneously. Water content within the sample was interpreted via the discrepancy of the cumulative amplitude of the *T*_2_ distribution between the standardized sample and the test samples. Detailed traxial shearing procedures were depicted in Li et al., (2021),^[^ ^47^ ^]^ whereas the NMR data analysis follows the method described by Ji et al., (2020).^[^ ^48^ ^]^

## Conflict of Interest

The authors declare no conflict of interest.

## Author Contributions

Y.L. designed the research and drafted the manuscript. Y.L. and M.Q. developed the indentation device and method. Y.Z. performed the indentation experiments and assisted in manuscript preparation. Z.Z. conducted molecular dynamics simulations. Y.Z. and Y.J. conducted the triaxial shear and low‐field NMR tests. Q.M., Y.L., and Y.Z. performed the XRD tests. Y.J. and Y.S. contributed to discussions on the nanoindentation, triaxial shear, and low‐field NMR tests. J.L. revised the manuscript. N.W. conceived the project and designed the method.

## Supporting information



Supporting Information

Supplementary Movie 1

Supplementary Movie 2

Supplementary Movie 3

Supplementary Movie 4

Supplementary Data

## Data Availability

The data that support the findings of this study are available on request from the corresponding author. The data are not publicly available due to privacy or ethical restrictions.
