# Supplementary material for: Stress‐Induced Melting Controlled Failure Mechanisms of Methane Hydrate
Source: Adv Sci (Weinh). 2025 Oct 31;13(10):e18367. doi: 10.1002/advs.202518367 (PMC12915162; doi:10.1002/advs.202518367)
Supplement: Supplementary file 1 — Supporting Information [file ADVS-13-e18367-s004.docx]

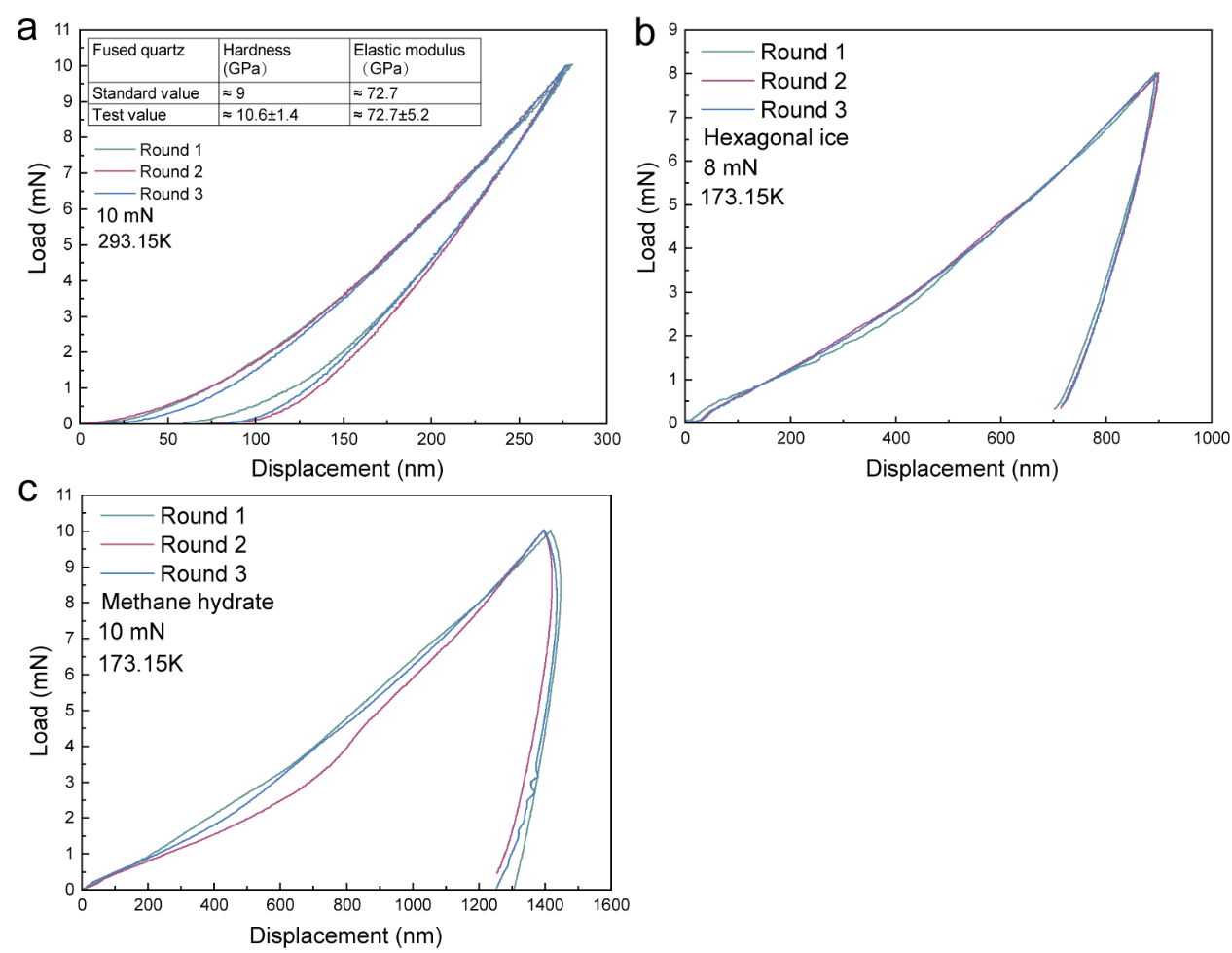


**Supplementary Fig. I | Repeated nanoindentation test results to ensure the reliability of the nanoindentation set-up. a**, Indentation curves for fused quartz under the temperature of 263.15K. Sub-figured table compares the intrinsic mechanical parameters (i.e., hardness, elastic modulus) of fused quartz between the test-inferred and the standard values. **b**, Repeated indentation tests for hexagonal ice under 173.15K, implying acceptable uncertainty errors. **c**, Repeated indentation tests for methane hydrate under 173.15K, which also implying acceptable uncertainty errors. Nanoindentation test was performed with >50 μm spacing between tests to ensure independence.


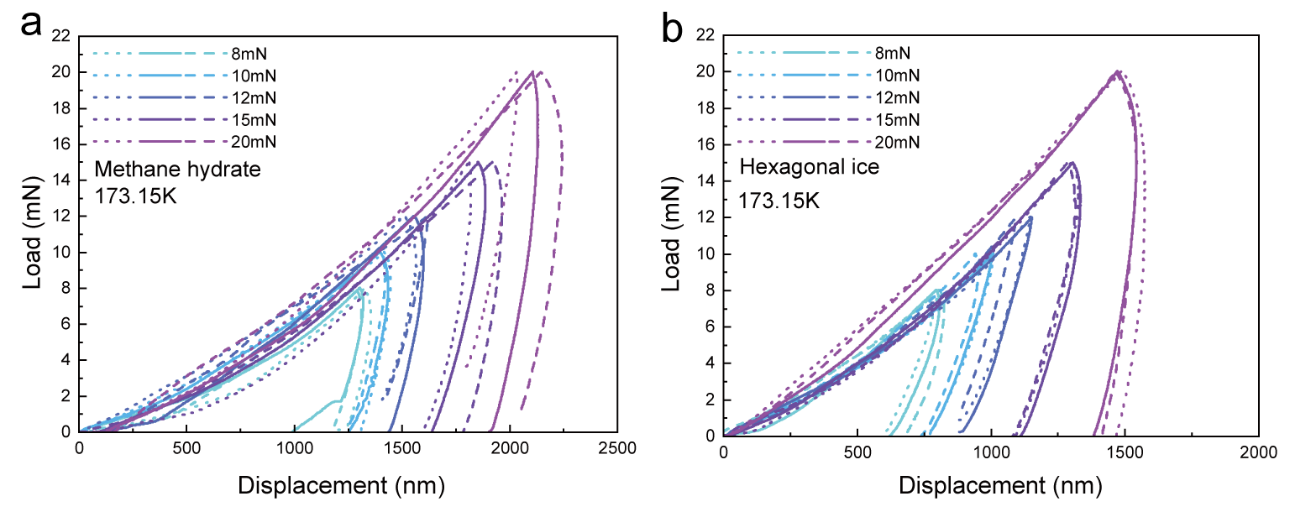


**Supplementary Fig. II | Nanoindentation curves for methane hydrate (a) and hexagonal ice (b) under the temperature of 173.15K.** At least 20 rounds of indentation were conducted under each loading, and only three of the results were displayed here. Loading was controlled within the range between 8 mN and 20 mN.


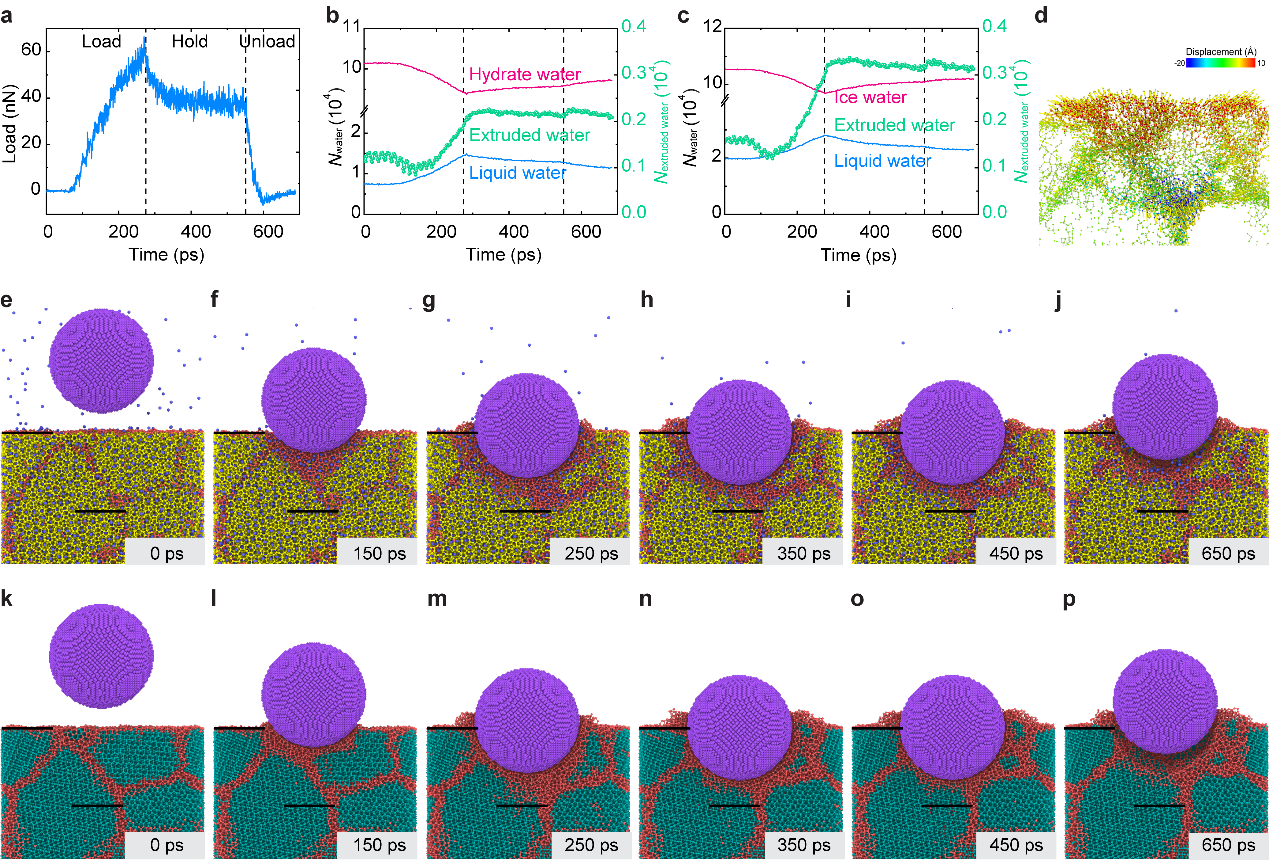


**Supplementary Fig. III | Nanoindentation mechanism analyzed by molecular dynamics simulations for the polycrystalline methane hydrate and hexagonal ice. a**, Indentation time-load curves for polycrystalline methane hydrate. **b**, The evolutionary of the number of hydrated water molecules (pink line), melted water molecules (blue line), and extruded water molecules (green line) during indenting polycrystalline methane hydrate. **c**, The evolutionary of the number of iced water molecules (pink line), melted water molecules (blue line), and extruded water molecules (green line) during indenting polycrystalline hexagonal ice. **d**, Displacement field of water molecules surrounding the indentation crater during holding. **e-j**, Snapshots taken from various stages of indentation for polycrystalline methane hydrate. **k-p**, Snapshots taken from various stages of indentation for polycrystalline hexagonal ice.

**Supplementary Video I： Visualization of the indentation processes for monocrystalline methane hydrate (note: Apparent upward molecular motion in hydrate simulations results from z-axis expansion due to methane release, ice systems show no comparable effect.)**

**Supplementary Video II： Visualization of the indentation processes for monocrystalline hexagonal ice**

**Supplementary Video III： Visualization of the indentation processes for polycrystalline methane hydrate**

**Supplementary Video IV Visualization of the indentation processes for polycrystalline hexagonal ice**
