# Supplementary material for: Stress‐Induced Melting Controlled Failure Mechanisms of Methane Hydrate
Source: Adv Sci (Weinh). 2025 Oct 31;13(10):e18367. doi: 10.1002/advs.202518367 (PMC12915162; doi:10.1002/advs.202518367)
Supplement: Supplementary file 6 — Supplementary Data [file ADVS-13-e18367-s005.docx]

**Key Points**

- Stress-application induces substantial regional melting of methane hydrate.
- Nanoindentation failure mechanisms for methane hydrate differs significantly with hexagonal ice.
- Hardness of methane hydrate is 261.4 ± 24 MPa, almost half of that for hexagonal ice (526.6 ± 62 MPa).
- Hexagonal ice is a poor proxy for methane hydrate from the mechanical perspective.
